# Points*See also Dr. C. Carleton Smith’s article, “Points,” in December number, 1886, page 432.

**Published:** 1887-02

**Authors:** Ad. Lippe

**Affiliations:** Philadelphia


### POINTS.*

AD. LIPPE, M. D., PHILADELPHIA.

*Dolichos pruriens.*—Invisible itching. The nightly rest is especially interrupted by a very distressing itching all over the body, without any perceptible or visible cause; it is especially indicated if this itching proceeds from liver diseases, and is indispensable when there is present *Icterus.*

*Symphitum officinale.*—Mechanical injuries, pains in broken bones, injuries of the eyes from blows, a snow-ball strikes the eye, an infant thrusts his fist into the mother's eye. Arnica is utterly useless in blows injuring the eye. Arnica will relieve

---

* See also Dr. C. Carleton Smith's article, "Points," in December number, 1886, page 432.

the soreness from injuries of soft parts, contusion of the brain, etc., but if the periosteum has also been injured, and the soft parts recovered from the bruises after giving Arnica, this soreness of the periosteum only becoming perceptible *afterward*, then Symphitum will relieve it at once.

*The Guiding Symptoms* of our materia medica, by Constantine Hering, are largely subscribed for under the management of F. A. Davis, 1207 Filbert Street, Philadelphia.

*Lac caninum.*—Pains and disorders *of all kinds*, changing from one side to the other; it was first observed in diphtheria, if first one, then the other, tonsil was affected, changing repeatedly, while Lachesis has as a characteristic symptom the spreading of the disease from the left to the right side, and Lycopodium the reverse, spreading from the right side to the left. Pulsatilla has wandering pains, but not characteristically changing sides only, but wandering over the whole body, attacking different parts irregularly, generally with swelling and redness of the joints. Sulphur has a similar shifting of gout. China, Arnica, Daphne ind., and Taxus have also shifting pains.

*Sticta pulmonalis.*—Will be found an indispensable remedy with stoppage of the nose, with an uncontrollable desire to blow the nose to no purpose. Even at night this blowing is continued, allowing of no sleep, and nothing is really blown from the nose. Nux vom. and Sambucus have stoppage of the nose, with discharge similar to that which often attacks infants (snifles). Lycopodium has stoppage of the nose at night, preventing breathing and sleep, with profuse discharges from the nose during the day.

*The Guiding Symptoms* of our materia medica, by Constantine Hering, are as indispensable to the library of a homœopathic physician as is Hahnemann's *Organon.* A library of a homœopathic physician without these works is as much of a mistake as the play of *Hamlet* with Hamlet left out.

*Causticum.*—Involuntary hard stool, worse during the daytime and when passing wind. Aloe has also involuntary passages of hard lumps of stool when walking. Coloc. has also involuntary formed stools. Causticum has pains in the bowels *after* a passage. Coloc. has the characteristic doubling-up pain *before* the stool.

*History* repeats itself. Dr. Roth in Paris undertook to sift the materia medica, struck out symptoms by the thousands, attempted to discredit Hahnemann, and he found willing lieutenants in his aim to annihilate our materia medica. Dr. Constantine Hering more than forty years ago wrote him down in a German quarterly, and in his despair he "lost his reason" and quit. The same attempt to annihilate our materia medica is made now. Hahnemann is discredited by a corresponding set of men, and history will repeat itself. The *Materia Medica* of the future and the threatened more complete farce will advance to the paper mill, and the designing pretenders will, like Roth, lose the little reason they ever had, if they ever had any at all.

